# Isolation and characterization of avian leukosis virus subgroup J associated with hemangioma and myelocytoma in layer chickens in China

**DOI:** 10.3389/fvets.2022.970818

**Published:** 2022-09-23

**Authors:** Lan Wu, Youjun Li, Xueyang Chen, Yuxin Yang, Chun Fang, Yufang Gu, Jing Liu, Xiongyan Liang, Yuying Yang

**Affiliations:** Laboratory of Animal Epidemic Diseases, College of Animal Science, Yangtze University, Jingzhou, China

**Keywords:** avian leukosis virus subgroup J, recombination, hemangioma, myelocytoma, layer chicken

## Abstract

A strain of avian leukosis virus (ALV) belonging to a new envelope subgroup J (ALV-J) emerged in 1988 as a new subgroup of ALV and spread rapidly throughout the world. Due to the infection and spread of ALV-J, the global poultry industry experienced a significant loss. Although the disease had been prevented and controlled effectively by culling domestic chickens in the infected zone, a few field cases of ALV-J infection were reported in China in recent years. This study was conducted to characterize the genome and analyze the lesions and histopathology of the ALV-J strain named HB2020, which was isolated from layer chickens in Hubei Province, China. The full-length proviral genome sequence analysis of ALV-J HB2020 revealed that it was a recombinant strain of ev-1 and HPRS-103 in the *gag* gene in comparison to ALV-J prototype HPRS-103. In the 3′-untranslated region (3'UTR) of the nucleotide sequence, there were found 205-base pairs (bp) deletion, of which 175 were detected in the redundant transmembrane (rTM) region. Besides, the surface glycoprotein gene *gp85* had five mutations in a conservative site, whereas the transmembrane protein gene *gp37* was relatively conserved. The animal experiments conducted later on this strain have shown that HB2020 can cause various neoplastic lesions in chickens, including enlarged livers with hemangiomas and spleens with white nodules. Additionally, as the exposure time increased, the number of tumor cells that resembled myelocytes in the blood smears of infected chickens gradually increased. These results indicated that HB2020 on recombination with ALV subgroup E (ALV-E) and ALV-J could induce severe hemangiomas and myelocytomas. This inference might provide a molecular basis for further research about the pathogenicity of ALV and emphasize the need for control and prevention of avian leukosis.

## Introduction

Avian leukosis virus (ALV) is an retrovirus that causes a variety of neoplastic diseases in poultry and has spread worldwide since its discovery in the early twentieth century (1). ALV infection in chickens could not only induce tumors and mortality but also cause a variety of diseases as a result of immunosuppression, which could affect their immunological response to vaccination and production performance (2). Furthermore, ALV has the potential to propagate vertically, causing injury to the progeny chickens and affecting the quality of commercial chickens.

The outbreak of avian leukosis subgroup J (ALV-J) among broiler breeders in 1988 was a major setback for the global chicken industry. The first evidence that ALV-J affects both layers and broilers appeared from the 2002 discovery of ALV-J in commercial layers in China (3). This discovery was significant as it laid the foundation for ALV-related research while also serving as a caution to the poultry industry, particularly layer farms.

ALVs are highly diverse RNA viruses with multiple subtypes that cause cancer in poultry (4). According to the properties of the viral envelope proteins, ALVs are divided into 10 subgroups (A-J), and the most recently identified subgroup is K (5).

ALVs isolated from chickens are classified into seven subgroups, such as ALV-A, ALV-B, ALV-C, ALV-D, ALV-E, ALV-J, and ALV-K (6). The genome of ALV is about 7.6–7.8 kb in length and possesses the classic slow transformation retrovirus genome structure 5'-R-U5-*gag*-*pol*-*env*-U3-R-3'. A variety of proteins are encoded by the *gag* gene, among which the major *gag* proteins include capsid protein (CA, *p27*), matrix protein (MA, *p19*), *p10*, nucleocapsid protein (NC, *p12*), and protease (PR, *p15*) (7). A previous study has revealed that a mutation in the *pol* gene could lead to increased reverse transcriptase activity, replication capacity, and increased vertical transmission capability of ALV-K; furthermore, this mutation is genetically stable, indicating the importance of the *pol* gene in ALV transmission (8).

The ALV *env* gene encodes envelope glycosylation protein, which is linked together with the surface glycoprotein subunit (SU) encoded by the *gp85* gene, and the transmembrane glycoprotein subunit (TM) encoded by the *gp37* gene into a rod-shaped dimer that is attached to the envelope surface, namely virus glycoprotein (9). *Gp85* is the ligand of the virus utilized to invade host cells, which also serves as the foundation for subgroup categorization (10). The significance of long terminal repeat (LTR) and *gp85* in the etiology of ALV-J has been confirmed by previous studies (11).

As a non-coding regulatory platform, the mRNA 3′-untranslated region (3′UTR) may influence mRNA stability and translation (12). The 3'UTR is critical to viral RNA replication as viral RNA molecules are typically replicated from the 3'-end to the 5'-end of the genome (13). Within the 3′UTR, ALV unique region 3 (U3) is unconservative (14). The typical transcriptional regulatory elements, namely CAAT, CArG, PRE, TATA, and Y boxes present in U3 were well conserved previously but began to alter in the most recent strain. The 11 bp deletion in U3 of ALV-J was suggested to be connected to the incidence of hemangioma in 2011 (15).

As ALV is retrovirus, it undergoes regular recombination, resulting in the emergence of new strains and subgroups. ALV-J is the most common novel subtype obtained from recombination, and this subtype was also suspected of being a recombinant virus (16). Until now, many recombinant strains have been discovered to be made up of various subpopulations, and it has been demonstrated that ALV infection ability can be altered following recombination (17). As a result, attention should be paid more to ALV recombination, and more in-depth studies should be conducted for disease prevention and control.

In this study, an ALV strain named HB2020 was isolated from infected layer chickens obtained from Jingzhou, Hubei province, which was identified as a J subtype subsequently using polymerase chain reaction (PCR). To analyze the sequence homology and divergence of HB2020, the whole genome was sequenced and compared with other ALV strains. As a result, the strain HB2020 was determined to be a hybrid of ALV-E and ALV-J. Following that, the embryos of 11-day-old chickens were intravascularly intravenously inoculated with the HB2020 strain, and their clinical symptoms were meticulously documented to study its pathogenicity. To understand the pathogenicity and variation of the ALV-J, these results are expected to provide a basis for further investigation. A theoretical framework for recent study on the pathogenicity and variation of the ALV-J strain circulated in China recently might be supplied by the foregoing experimental results.

## Methods

### Clinical examination

In April 2020, a large number of layer chickens belonging to a commercial indigenous chicken farm in Hubei province of China displayed thinness and decreased egg production rate, and some chickens passed away before they attained 60 days of age. The chicken flocks' morbidity and mortality were 14.9 and 14.6%, respectively. From the farm mentioned previously, five 20-weeks-old layer chickens with substantial clinical symptoms in egg production were submitted to our laboratory for further diagnosis.

### Virus isolation

Virus isolation was performed using DF-1 chicken fibroblast cell line without endogenous ALV gene to exclude endogenous ALV interference. The DF-1 cells were planted in a six-well plate containing Dulbecco's Modified Eagle Medium (DMEM, Invitrogen^®^) with 10% fetal bovine serum (FBS, Gemini^®^). At 70% confluence, the DF-1 cells were co-cultured with filtered liver homogenates obtained from the positive sample and incubated for 2 h at 37°C. The cells were then continuously grown in DMEM containing 1% FBS in an incubator with 5% CO_2_ at 37°C. The supernatant from infected cells was collected after three blind passes to identify ALV using an ALV group-specific antigen (p27) enzyme-linked immunosorbent assay (ELISA, IDEXX^®^). Positive cells were collected for proviral DNA extraction employing the Genomic DNA Extraction Kit (GK0222, Generay Biotech. Co. Ltd., Shanghai, China) based on the manufacturer's instructions, subsequently eluted with 50 μL of DNase-free water, and kept at −80°C. The DNA extracted from P27-positive cells was identified using PCR to exclude Marek's disease virus (MDV) (18) and reticuloendotheliosis virus (REV) (19) (Table 1). The MDV and REV cases used as positive controls in PCR were archived in our laboratory (20, 21). Primer pairs A/B/J/K-F/R (Table 1) were used to detect ALV and define its subgroup (22, 23). The median tissue culture infectious dose (TCID_50_) in these cells' supernatant was calculated by the Reed-Muench method.

**Table 1:** Sequences of the primers for chicken common neoplastic diseases.

**Primers**	**Sequences**	**PCR product size**
MDV-F	5'-GGATGAGGTGACTAAGAAAG-3'	856 bp
MDV-R	5'-GGATGAGGTGACTAAGAAAG-3'	
REV-F	5'-GGATGAGGTGACTAAGAAAG-3'	204 bp
REV-R	5'-GGATGAGGTGACTAAGAAAG-3'	
ALV-F	5'-CGGAGAAGACACCCTTGCT-3'	
ALV-AR	5'-GCATTGCCACAGCGGTACTG-3'	715 bp
ALV-BR	5'-GTAGACACCAGCCGGACTATC-3'	515 bp
ALV-JR	5'-CGAACCAAAGGTAACACACG-3'	422 bp
ALV-KR	5'-TTGCGGCCTGGACCAATC-3'	535 bp

### Phylogenetic and recombination analysis

The entire proviral genome of HB2020 was amplified by a segmented PCR experiment (Table 2), using the template DNA extracted from the ELISA-positive cells as previously described. The whole proviral genomic DNA of HB2020 was sequenced and spliced using DNASTAR software. Sequence alignment with other ALV strains was performed using the DNASTAR software and the NCBI BLAST tool. Major genome-wide genes, such as *gag, pol, gp37, gp85*, and LTR, were compared with other ALV strains (Table 3). In molecular evolutionary genetics analysis version 6.0 (MEGA 6), the neighbor-joining approach was used to perform phylogenetic analysis with 1,000 bootstrap repetitions. Recombination detection program version 5 (RDP5) and SimPlot software (V3.5.1) were used to examine the possible recombination events for the entire nucleotide sequence of HB2020; the Kimura two-parameter model was used to map them, and 100 bootstrap replicates were employed to evaluate the reliability of the recombination event. The parental threshold cut-off value was set above 70%.

**Table 2:** Sequences of the segmented primers.

**Primers**	**Sequences**	**PCR product size**
AF	5'- ACGCGTTGTAGTCTTATGCAATGCTCTT−3'	2 500 bp
AR	5'- GCATGGGAATTCCCCCTCCTATC−3'	
BF	5'- ACGCGTTGCGAATTCCCATGCGAAAATCT−3'	2 700 bp
BR	5'- GTCGACTCATCCTTTTGGGTGATGT−3'	
CF	5'- AGAAGGACCCCCGGTTAAAATACGAAT−3'	2 700 bp
CR	5'- GTCGACTGAAGCCATCCGCTTCATGCAGGT−3'	

**Table 3:** ALV strains used for comparison of the sequence.

**Isolate**	**Accession no**.	**Origin**	**Year**	**Subgroup**	**Tumor phenotype**
RAV-1	MF926337	USA	1980	A	N/A
MAV-1	L10922	FRA	1993	A	N/A
MAV-2	L10924	CAN	1993	B	N/A
Prague C	J02342	USA	1977	C	N/A
RSV Schmidt-Ruppin D	D10652	JPN	1922	D	N/A
ev-1	AY013303	USA	2000	E	N/A
GDFX0601	KP686142	CHN	2015	K	N/A
JS11C1	KF746200	CHN	2013	K	ML
JL093-1	JN624878	CHN	2009	J	HE&ML
GD1109	JX254901	CHN	2011	J	HE&ML
GD19GZ01	MN893850	CHN	2019	J	HE
GDQY1201	JX423792	CHN	2012	J	ML
GDYH-B1	MK940585	CHN	2018	J	HE
HPRS103	Z46390	UK	1988	J	HE
JS09GY3	GU982308	CHN	2009	J	HE&ML
JS09GY6	GU982310	CHN	2009	J	HE&ML
JS-nt	HM235667	CHN	2003	J	HE
NX0101	DQ115805	CHN	2001	J	ML
PDRC-59831	KP284572	USA	2007	J	HE&ML
SCAU11-H	KC149972	CHN	2011	J	HE&ML
SCAU11-XG	KC149971	CHN	2012	J	HE&ML
SCAU-HN06	HQ900844	CHN	2007	J	HE
SDAU1701	KY980657	CHN	2017	J	HE

N/A, data not available; ML, means myeloma leukosis; HE, means hemangioma leukosis.

### Analysis of the pathogenicity

Sixty embryonated specific pathogen-free (SPF) eggs (Specific Pathogen Free Avian Supplies) were split into two groups (an infection group and a negative control group) randomly, each comprising 30 embryonated eggs. At the age of 12 embryonic days, eggs in the infection group were intravenously inoculated with ALV-J of 10^4^ TCID_50_ (24). The rest of the cohort was subjected to DMEM inoculation in the same manner. The chicks that attained the age of 3 days were vaccinated against MDV. Thereupon, PCR amplification of ALV-J was performed with H5/H7 primer to detect if the chicks were infected with ALV-J (Table 4). To generate blood smears, blood was withdrawn from each chick at the age of 4, 10, and 16 weeks throughout the chicken cultivation phase; a drop of fresh blood was taken and smeared evenly on a clean slide and stained with Wright-Giemsa (BASO BA-4017, Zhuhai, China) for examination. All chicks were subjected to bloodletting, and a thorough necropsy was performed on them at the age of 21 weeks.

**Table 4:** Sequences of the primers for ALV-J.

**Primers**	**Sequences**	**PCR product size**
H5	5'-GGATGAGGTGACTAAGAAAG-3'	545 bp
H7	5'-CGAACCAAAGGTAACACACG-3'	

## Results

### Virus isolation and identification

**Figure 1:**
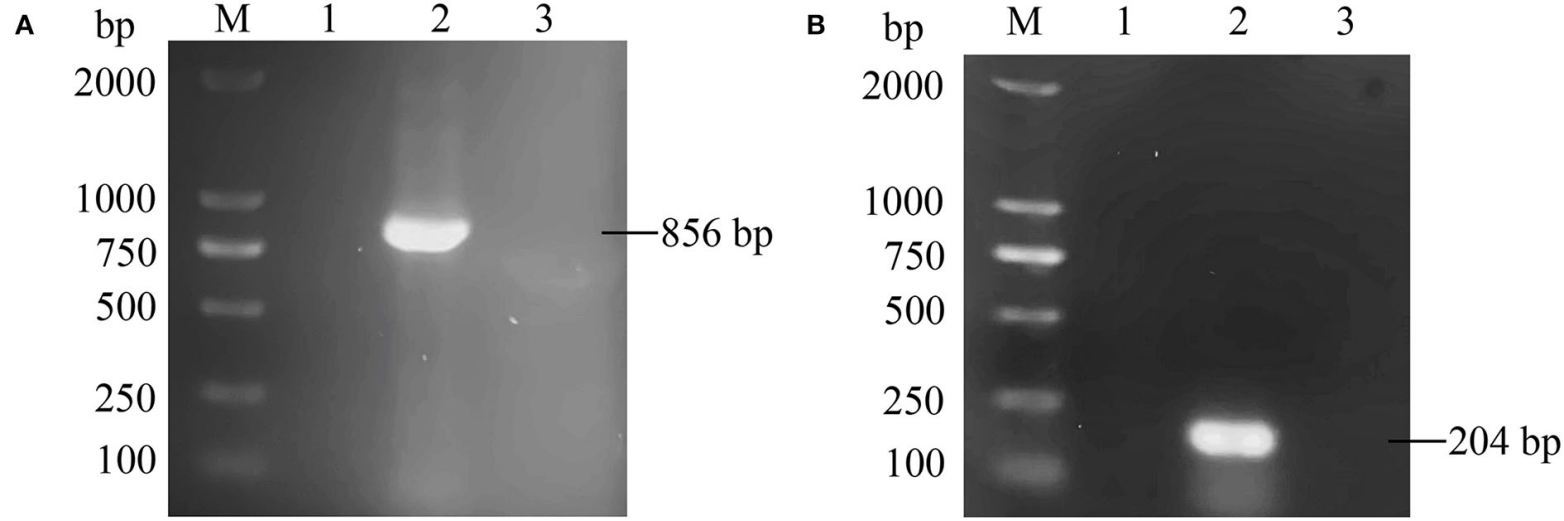
**(A)** The PCR result with primer MDV-F/R. M:maker, 1: negative control, 2: positive control, 3:HB2020. **(B)** The PCR result with primer REV-F/R. M:maker, 1: negative control, 2: positive control, 3:HB2020.

**Figure 2:**
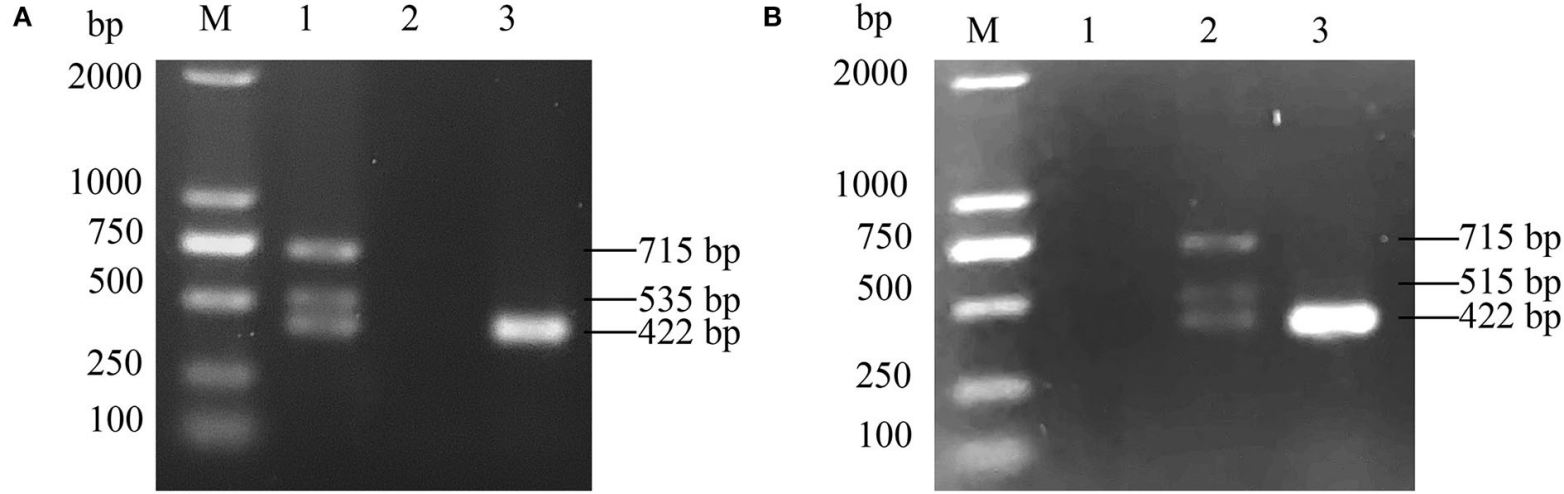
**(A)** The PCR result of A/J/K Multi-PCR. M:maker, 1: positive control, 2: negative control, 3:HB2020. **(B)** The PCR result of A/B/J Multi-PCR. M:maker, 1: negative control, 2: positive control, 3:HB2020.

An ALV-J strain, named HB2020 (GenBank accession number: ON840093), was isolated and identified from the affected layer chickens using DF-1 cell co-culture and a PCR assay. Seven days after inoculation, the supernatants of infected DF-1 cells yielded a positive result using the ALV group-specific antigen (p27) ELISA (IDEXX), indicating the presence of ALV in the samples. PCR with the primers for MDV and REV exhibited negative results (Figure 1). The multi-PCR for A/B/J and A/J/K generated a 422 bp fragment (Figure 2), indicating that HB2020 is ALV-J. The Reed-Muench method determined that each 0.1 mL of DF-1 cellular supernatant had 10^4^ TCID_50_.

### Molecular characterization of the genome

**Figure 3:**
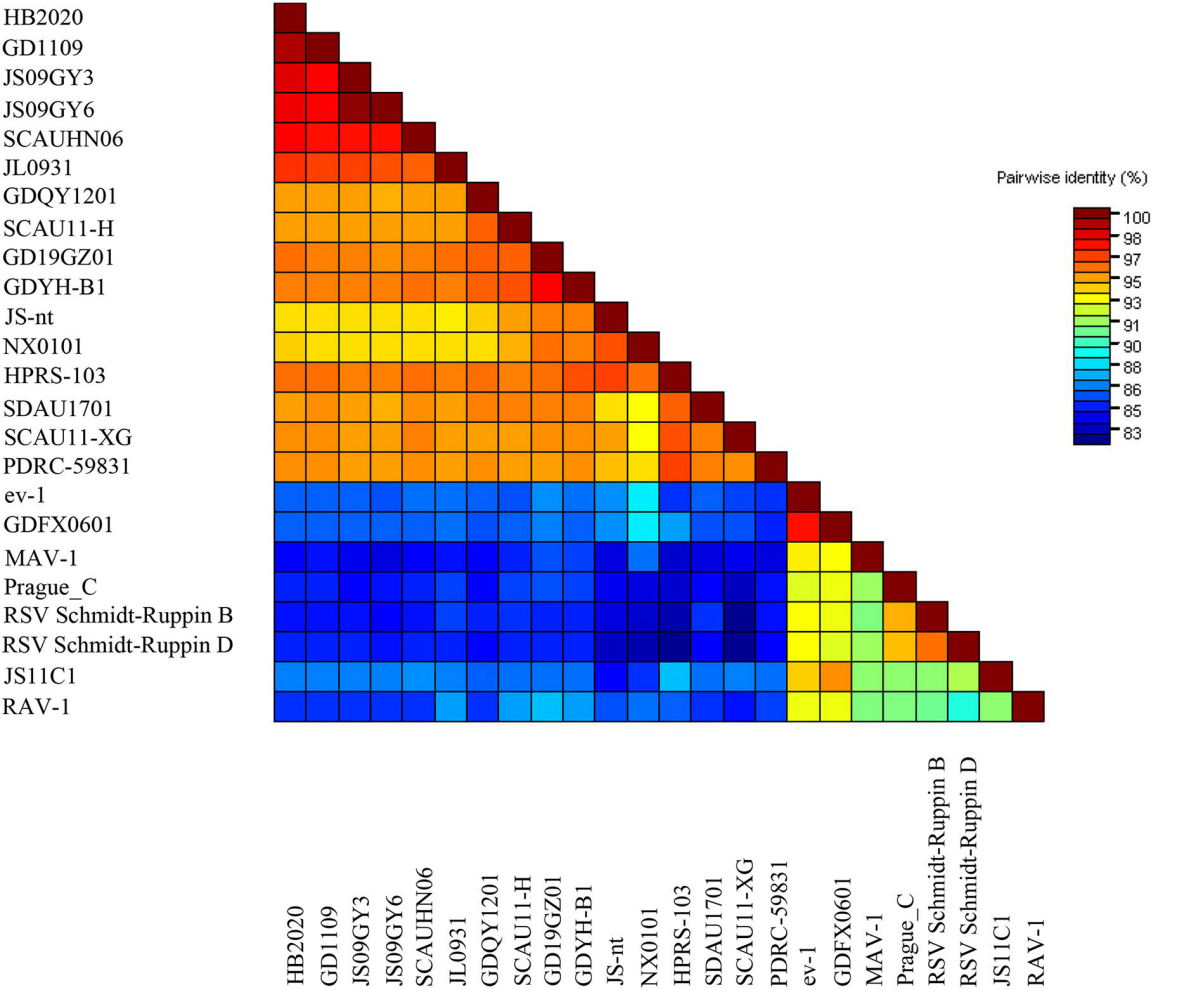
The pairwise identities plot of the entire gene for the HB2020 isolates reported in this study (red colored), aligned by ClustalW and displayed by the Sequence Demarcation Tool (SDT) software.

**Figure 4:**
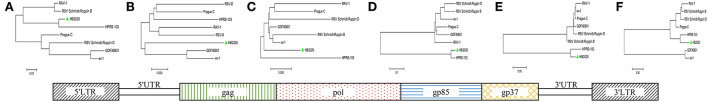
ALV isolate HB2020 and other ALVs were compared in terms of segmental sequences. The genome sequences were divided into functional areas, and each segmental sequence underwent sequence alignment. The genome structure of the isolate HB2020 is shown in the bottom box. Phylogenetic tree: **(A)** 5'LTR. **(B)** gag. **(C)** pol. **(D)** gp85. **(E)** gp37. **(F)** 3'LTR. The Neighbor-Joining method with 1,000 bootstrap replicates was used to infer the evolutionary relationships of each fragment (MEGA 6).

**Figure 5:**
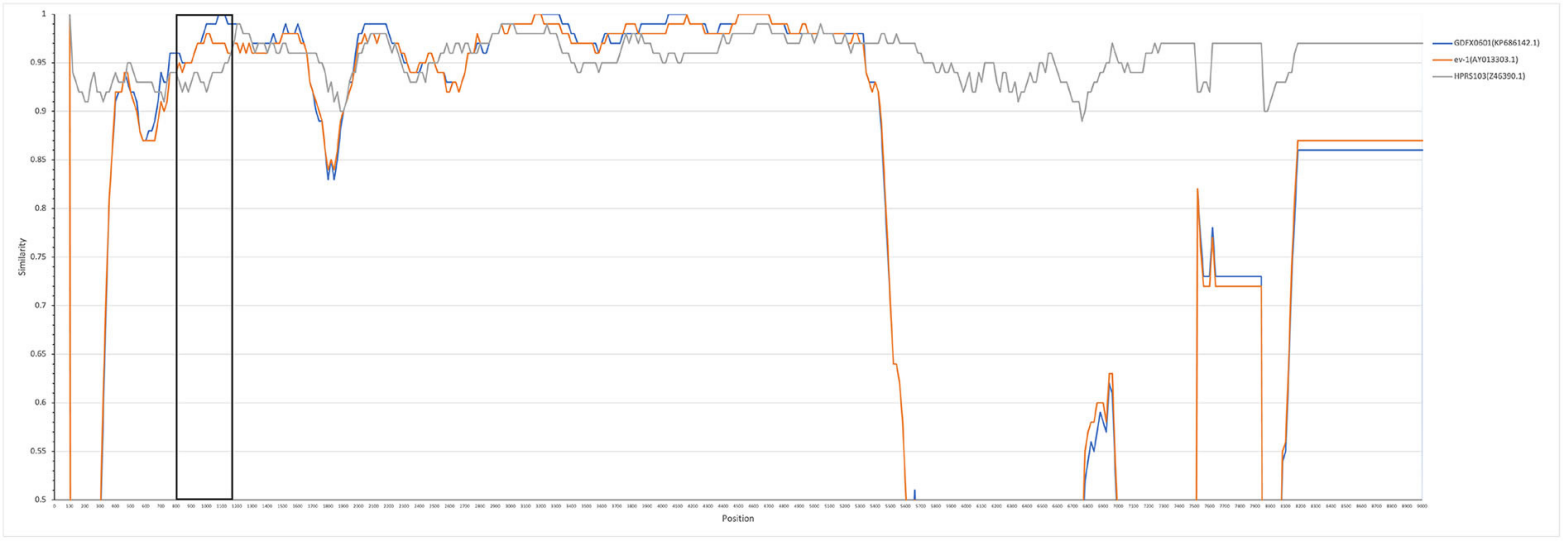
HB2020 recombination analysis SimPlot software (V3.5.1) was used to analyze recombination events, which were then mapped using the Kimura (2-parameter) model. The reliability of the recombination event was assessed using a 100-bootstrap, and a parental threshold of > 70% was chosen as the cut-off value.

The entire genome of HB2020 was 7471 bp long, assembled by the SeqMan program of DNASTAR software. In the heat map, HB2020 clustered in the same region as the other ALV-J (Figure 3). Phylogenetic analysis of the *gag* genes revealed two distinct branches: *gag* regions of HB2020 clustered with ev-1, and the ALV prototype HPRS-103 clustered with Rous sarcoma virus (strain Prague C) (RSV-PrC) and Schmidt-Ruppin D strain of RSV (SR-RSV-D(H) (Figure 4B). The genes and functional regions (LTR, *pol, gp85*, and *gp37*) of HB2020 and HPRS-103 were found in the same branch in their respective phylogenetic trees (Figure 4). The analysis of recombination events revealed that the major recombination sites of HB2020 and ev-1 were located in *gag* regions (Figure 5).

### Sequence analysis of the *gp85* gene

**Figure 6:**
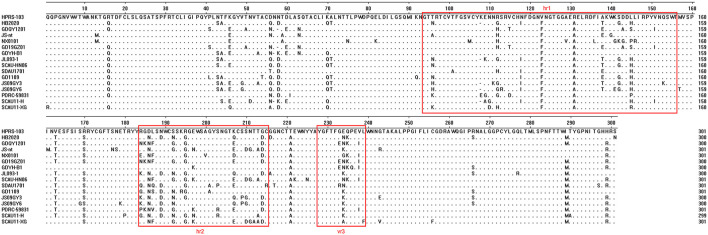
Comparison of the amino acid sequences of gp85 from ALV-J isolates and the HB2020 strain. The inferred gp85 was compared to other ALV strains. The colored boxes represent the gp85 hr1, hr2, and vr3 regions.

Mutations were more likely to occur in the hypervariable regions 1 (hr1), 2 (hr2), and variable region 3 (vr3) of HB2020, with 32 mutations found in the *gp85*-encoded surface glycoprotein (SU) (Figure 6). Five (N58, K70, A71, K108, and N112) of the nine (N58, D60, K70, A71, K108, N112, N113, N121, and R272) frequent mutations in ALV-J were discovered in SU of HB2020. In addition, mutations in three amino acids (E109, R112, and N118) were found in the hr1 region of ALV-J that causes myeloid leukosis (Figure 6). The mutation of amino acid Q235 in the vr3 of HB2020 had not been reported in viruses that induce both myeloid and hemangiomatous avian leukosis cited in this study (JL093-1, GD1109, JS09GY3, JS09GY6, PDRC-5983, SCAU11-H, SCAU11-XG) (Figure 6).

### Sequence analysis of the 3'UTR gene

**Figure 7:**
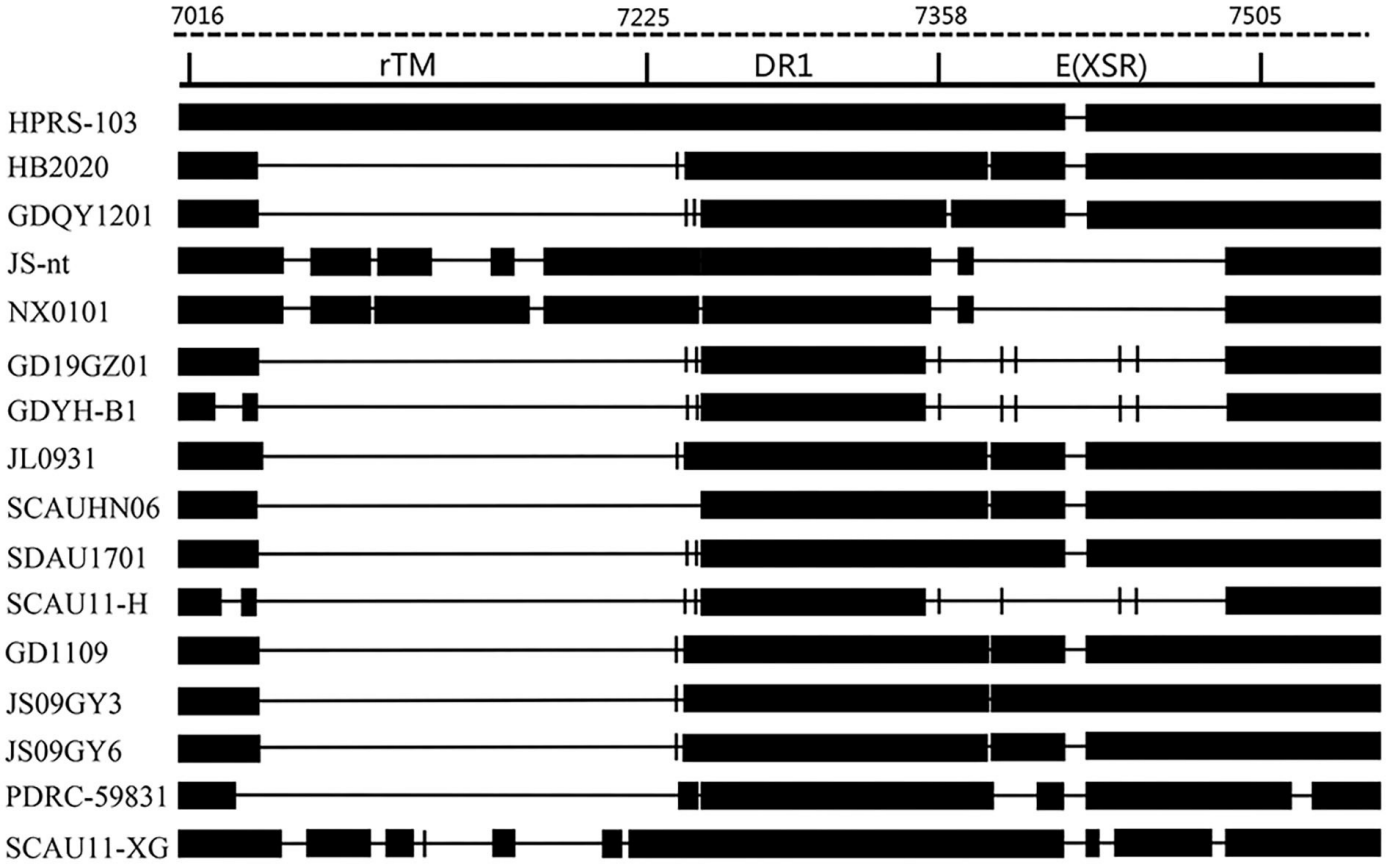
Comparison of the nucleotide and important regulatory elements of the HB2020 and other ALVs' 3'UTR regions.

**Figure 8:**
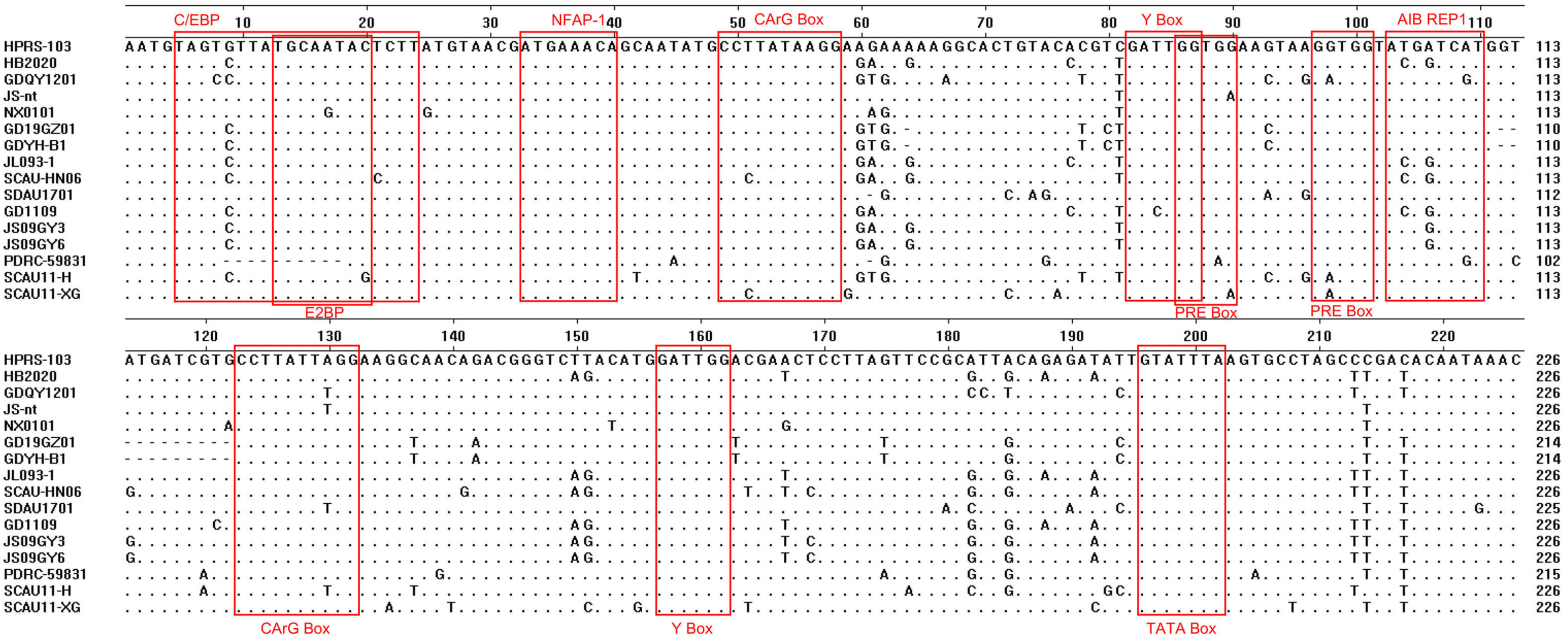
Comparative analysis of the U3 region of different ALV-J strains.

The 3'UTR of HB2020 was 542 bp long, containing the rTM region, direct repeat 1 (DR1), and the E element. There was a 175 bp deletion in rTM of HB2020, which was consistent with ALV-J (JL093–1) isolated from a hemangioma case and numerous ALV-J strains (GD1109, JS09GY3, and JS09GY6) that potentially induced both hemangiomas and myeloma (Figure 7). The DR1 was exceedingly conservative in these referenced ALV-J, and the complete initial E element of HB2020 was 174 bp long (Figure 7). The SoftBerry NSITE online service analysis system was employed to analyze the transcriptional regulatory elements; the result revealed that in the U3 region of the HB2020 genome, the transcriptional regulatory elements, including C/EBP, E2BP, CArG box, and Y box, were substantially conserved in comparison to HPRS-103 strain (Figure 8). In contrast to the HPRS-103 strain, the AIB REP1 transcription factor of HB2020 possessed two mutant bases (Figure 8).

### Gross and histological lesions

After incubation, 24 infected chicks survived, in comparison to 26 survivors in the uninfected control group. Blood cells' DNA was extracted from two chickens of the negative control group chosen randomly and tested using PCR with the H5/H7 primer. The result revealed that there were no positive findings, indicating that the chicken embryos had not been infected with ALV-J. At 4 weeks post infections (wpi), all of the chickens in the infected group were positive when PCR was performed with the H5/H7 primer, and four chickens in the infected group exhibited clinical symptoms, such as loss of appetite, listlessness, and diarrhea. At 10 wpi, the symptoms worsened (Table 5). The first instance of the clinical tumor was detected at 16 wpi, and nine further cases were detected at 20 wpi.

**Table 5:** The cumulative morbidity and mortality of the HB2020 infected chickens.

**Time (Wpi)**	**Cumulative morbidity**		**Cumulative mortality**	
	**HB2020**	**Control**	**HB2020**	**Control**
4	16%	0%	0%	0%
10	50%	0%	16%	0%
16	60%	0%	46%	0%
20	100%	0%	58%	0%

**Figure 9:**
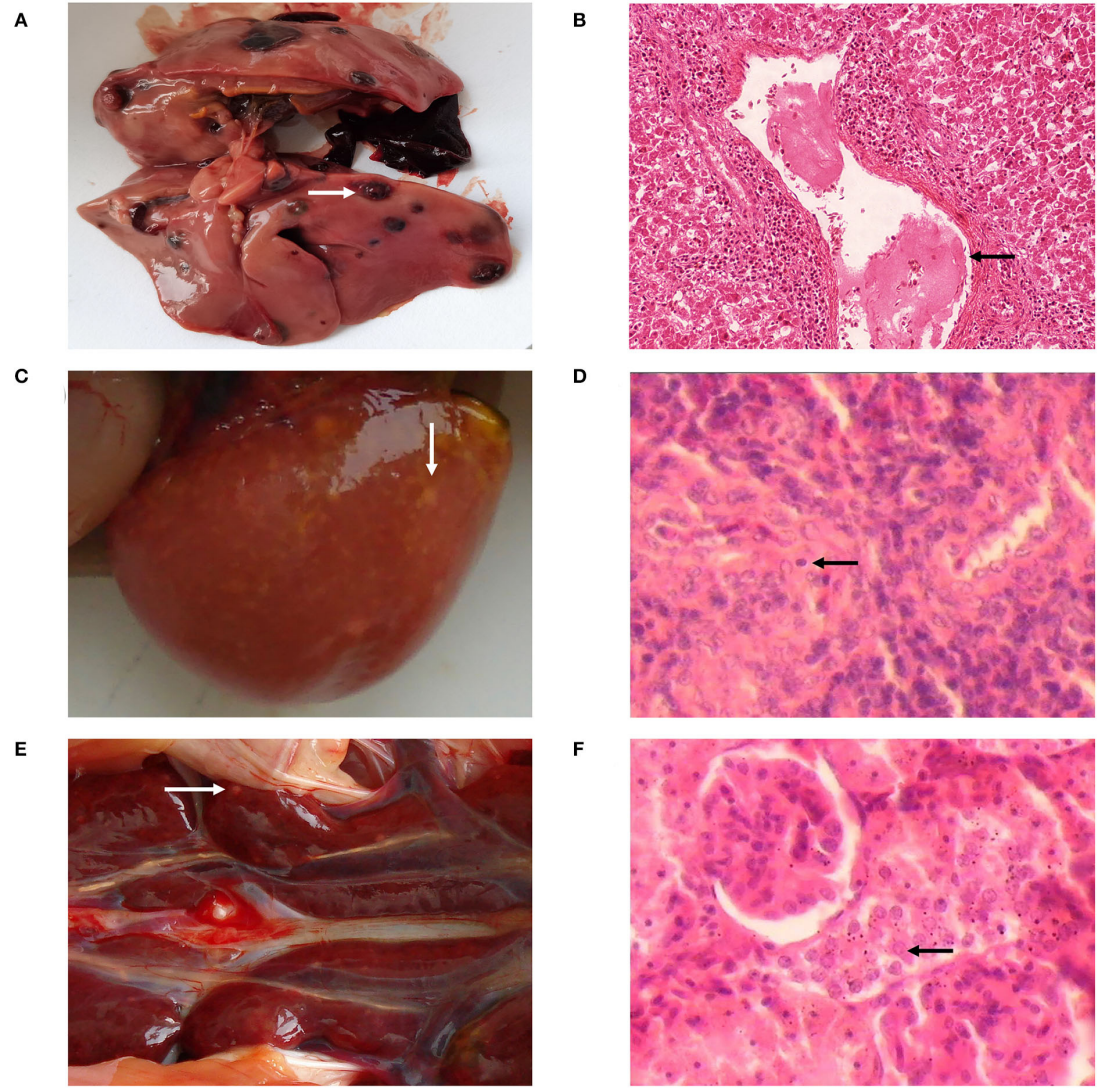
Anatomical and histological lesions. **(A)** Hemangioma on the liver surface. **(B)** Vascular endothelial hyperplasia, HE, 40×. **(C)** Lesions on the surface of the spleen. **(D)** Proliferation of capillary vessels, HE, 400×. **(E)** Kidney enlarged with multifocal lesions. **(F)** Kidney cells without nucleolus, HE, 400×.

The dead affected chickens were immediately examined by necropsies. Most layer chickens in the infected group exhibited typical necropsy lesions of avian leukosis. Numerous hemangiomas in their dark red liver were spotted (Figure 9A), and the spleen was grossly enlarged with numerous white necrotic lesions of varying sizes (Figure 9C). The kidneys were swollen and covered with white nodules (Figure 9E). Under the microscope, vascular endothelial cells were hyperplastic and clustered in the liver (Figure 9B); splenic vascular endothelial cells were hyperplastic and clustered, with partial lymphocyte nuclei being absent (Figure 9D), and the kidney revealed a large number of myeloid tumor cells (Figure 9F).

### Blood analysis

**Figure 10:**
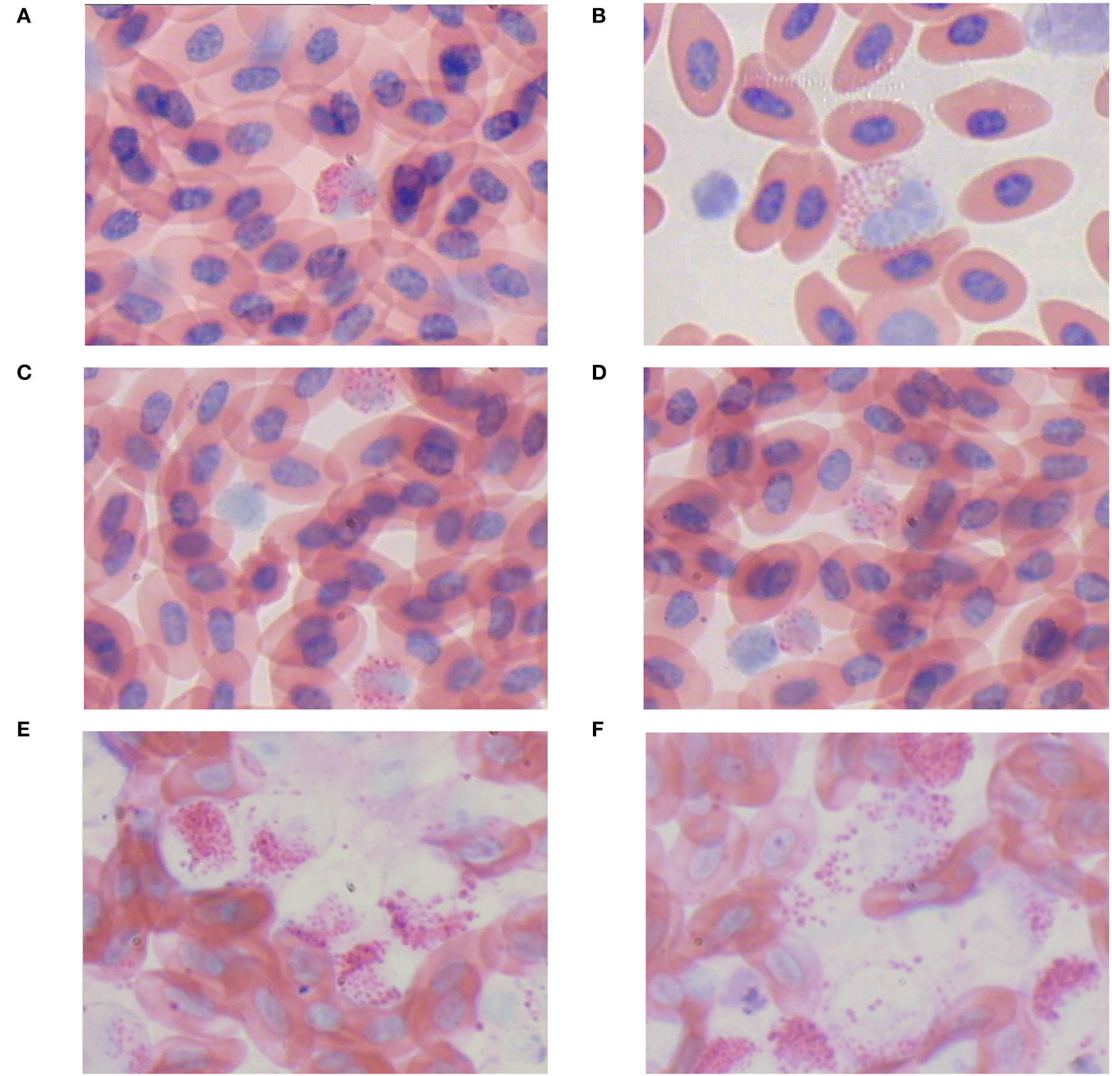
Blood swears (ME, 400×). **(A,B)** An myelocyte-like tumor cell. **(C,D)** The number of myelocyte-like tumor cell was increased. **(E,F)** The lysis of myelocyte-like tumor cells.

Blood smears revealed the presence of a few myeloid tumor cells in the blood of four-week-old chickens of the infected group (Figures 10A,B). Myeloid tumor cells were large and spherical, with a round or oval nucleus on one side of the cell and a substantial number of acidophilic granules in the cytoplasm (Figures 10A,B). An increase in myeloid tumor cells was observed in the blood smears of 10-week-old chickens (Figures 10C,D). A large number of myeloid tumor cells were observed microscopically in the blood smears of 16-week-old chickens (Figures 10E,F).

## Discussion

In 1999, it was first reported that some broiler flocks from China had been infected with ALV-J. Subsequently, ALV-J spread rapidly in Chinese chicken flocks, causing huge losses to the Chinese chicken industry. This, along with the continued expansion of the virus' host range and the emergence of new strains with novel symptoms and lesions induced, sparked people's widespread concern (25). Therefore, an Eradication Program for avian leukosis was implemented in China about 10 years ago, which effectively prevented and controlled the disease. However, since 2017, there has been another ALV-J infection outbreak in several provinces of China, leading to an increase in serious tumor diseases and a sharp increase in mortality (26). Therefore, in this study, a strain of ALV-J was isolated from layer chickens, and some typical ALV cases were successfully replicated in white-feathered chickens. We discovered that liver with hemangiomas, kidney and spleen enlargement with white nodules to be common symptoms in the reproduced cases. Some chickens had numerous hemangiomas and bony nodules. The symptoms of the cases reproduced in this study were more severe than the clinical symptoms of HPRS-103 (27), suggesting that HB2020 is more virulent. The clinical symptoms as well as the various tumor types induced by HB2020 indicated that HB2020 might possess different molecular characteristics.

In 2009, approximately 70% of avian leukosis cases in China were infected with ALV-J (28). ALV-J is another subtype with a high mutation rate; the *env* gene of ALV-J shares 80–85% homology with the nucleotide sequences of its subgroups but only 40% homology with the nucleotide sequences of other subgroups (29). The HB2020's *gag* gene was closely related to ev-1 and GDFX0601 based on the level of sequence similarities, but the remaining genes and regions were on the same branches as HPRS-103 in their respective trees, therefore we speculated that the virus isolated, HB2020, was a recombinant strain belonging to subgroup J that had an ALV-E-like *gag*. The same recombination in *gag* regions was also detected in ALV-J strain LH20180301, isolated from a commercial farm in Shandong province of China (30). This mutation emerged very recently, and it may represent a new direction of variation in ALV-J isolated from China.

The envelope glycosylation protein (ENV) is composed of *gp85*-encoded surface glycoprotein and *gp37*-encoded transmembrane glycoprotein, and it is closely related to the virus' antigenicity, tumorigenic type, and host range (31). We confirmed that the HB2020 strain isolated in this study had some variation in ENV compared to all other ALV-J strains. The results revealed that the mutations of HB2020 were primarily concentrated in the vr3, hr1, and hr2 regions of the SU in *env* gene. Furthermore, we found that the genotypes of ALV-J *gp85* that cause various pathological symptoms follow a pattern. Three specific amino acid mutations (E109, R112, and N118) were found in the SU of ALV-J that cause hemangiomas. However, a unique amino acid mutation Q235 could only be observed in the SU of the ALV-J strains that cause myeloid leukosis. This phenomenon indicated that these gene mutations might be associated with infectivity and pathogenicity; however, the mechanism of this phenomenon remains unclear. So far, several studies have shown that the sequences spanning amino acids 38 to 131 and 159 to 283 of ALV-J SU could influence the binding efficiency of chicken Na+/H+ Exchanger 1 (chNHE1), which was important to receptor binding and viral entry for N6, N11 sites and C3, C9 cysteines of ALV-J *gp85* (32). The mechanism of the phenomenon observed in this study requires further investigation.

The 3' UTR of ALV is frequently mutated and deleted (33). Since 2012, a few ALVs with a deletion of 205 bp in the 3' UTR have been discovered in China, indicating that these viruses are more oncogenic (34, 35). The HB2020 also had 205 bp deletions in the 3'UTR, and animal testing revealed that it was highly pathogenic, and could cause systemic tumors, which was consistent with previous findings. It had an affinity for the LTR's U3 region and had been implicated in the propagation of the avian retroviruses (36). Many important protein binding motifs have been identified in the U3 region, including COAAT/enhancer, CArG box, and Y box elements. C/EBP, E2BP, CArG box, AIB REP1, and Y box could be found in the U3 region of HB2020. It is note worthy that AIB REP1 binding sites have been found in all myeloid leukosis (ML) ALV-Js, including HB2020. However, the AIB REP1 in the U3 region of HB2020 had two mutant bases. The AIB REP1 gene has been linked to the vascular invasion of hepatocellular carcinoma in humans (37). In this study, a lot of hemangiomas were observed in the chicken's liver with the most obvious pathological symptoms; perhaps this phenomenon was related to the AIB REP1 mutation and the role of AIB REP1 in ALV-induced tumorigenesis requires further investigation.

So far, ALV is still spreading all over the world. The molecular biological analysis and tumorigenicity experiment of HB2020 might help to monitor the mutation tendency and study the prevalence of ALV-J, and guide preventive actions.

## Data availability statement

The datasets presented in this study can be found in online repositories. The names of the repository/repositories and accession number(s) can be found in the article.

## Ethics statement

The animal study was reviewed and approved by the Academic Committee of the College of Animal Science, Yangtze University.

## Author contributions

All authors listed have made a substantial, direct, and intellectual contribution to the work and approved it for publication.

## Funding

This work was supported by the National Nature Science Foundation of China (31972646), State Key Laboratory of Veterinary Biotechnology Foundation, China (SKLVBF201708), and Scientific Research Project of Education Department of Hubei Province (B2019028).

## Conflict of interest

The authors declare that the research was conducted in the absence of any commercial or financial relationships that could be construed as a potential conflict of interest.

## Publisher's note

All claims expressed in this article are solely those of the authors and do not necessarily represent those of their affiliated organizations, or those of the publisher, the editors and the reviewers. Any product that may be evaluated in this article, or claim that may be made by its manufacturer, is not guaranteed or endorsed by the publisher.
